# Atrial fibrillation: real-life experience of a rhythm control with electrical cardioversion in a community hospital

**DOI:** 10.1186/s12872-024-03885-0

**Published:** 2024-04-17

**Authors:** Artemiy Okhotin, Maxim Osipov, Vasilij Osipov, Anton Barchuk

**Affiliations:** 1 ITMO University, Kronverkskiy Prospekt, 49, 197101 St. Petersburg, Russia; 2 Tarusa Hospital, K. Libknekhta ulitsa, 16, 249100 Tarusa, Russia; 3 Institute for Interdisciplinary Health Research, European University at St. Petersburg, Shpalernaya Ulitsa 1, 191187 St. Petersburg, Russia

**Keywords:** Atrial fibrillation, Electrical cardioversion, Community hospital

## Abstract

**Background:**

Atrial fibrillation is the most prevalent sustained cardiac arrhythmia. Electrical cardioversion, a well-established part of the rhythm control strategy, is probably underused in community settings. Here, we describe its use, safety, and effectiveness in a cohort of patients with atrial fibrillation treated in rural settings.

**Methods:**

It is a retrospective cohort study. Data on all procedures from January 1, 2016, till December 1, 2022, in Tarusa Hospital, serving mostly a rural population of 15,000 people, were extracted from electronic health records. Data on the procedure’s success, age, gender, body mass index, comorbidities, previous procedures, echocardiographic parameters, type and duration of arrhythmia, anticoagulation, antiarrhythmic drugs, transesophageal echocardiography, and settings were available.

**Results:**

Altogether, 1,272 procedures in 435 patients were performed during the study period. The overall effectiveness of the procedure was 92%. Effectiveness was similar across all prespecified subgroups. Electrical cardioversion was less effective in patients undergoing the procedure for the first time (86%, 95% CI: 82-90) compared to repeated procedures (95%, 95% CI: 93-96), OR 0.39 (95% CI: 0.26-0.59). Complications were encountered in 13 (1.02%) procedures but were not serious.

**Conclusions:**

Electrical cardioversion is an immediately effective procedure that can be safely performed in community hospitals, both in inpatient and outpatient settings. Further studies with longer follow-up are needed to investigate the rate of sinus rhythm maintenance in these patients.

**Supplementary Information:**

The online version contains supplementary material available at 10.1186/s12872-024-03885-0.

## Background

Atrial fibrillation (AF) is the most common sustained cardiac arrhythmia with significant associated morbidity and mortality [1]. Its prevalence is growing along with the ageing of the population. AF is mutually connected with congestive heart failure, their co-existence being referred to as heart failure and AF dual epidemic [2]. Treatment of AF may be comprehended via the ABC pathway delineated in ESC Guidelines: anticoagulation/avoid stroke, better symptom control and detection and management of cardiovascular risk factors and concomitant disease. Better symptom control can be achieved through antiarrhythmic therapies, rate control medications, acute treatment of paroxysms with electrical and pharmacological cardioversion, and radiofrequency ablation of arrhythmia triggers and substrate [3]. Two primary strategies for the treatment of AF are rate and rhythm control. In the rate control strategy, the arrhythmic substrate and triggers *per se* are left unaddressed, while a safe reduction in ventricular response rate becomes the primary goal of treatment. Within the rhythm control strategy, treatment is directed at restoring and maintaining sinus rhythm. Restoration of sinus rhythm can be achieved by pharmacologic means or by electrical cardioversion - a procedure involving the application of a short burst of direct electric current to the atrial myocardium under deep sedation or general anaesthesia.

The management paradigm of AF has significantly changed over time. Introducing direct current electrical cardioversion and antiarrhythmic drugs enabled physicians to restore and maintain sinus rhythm, albeit with moderate efficacy [4]. Still, earlier randomised clinical trials, RACE, and AFFIRM, have not shown mortality benefit, which curbed enthusiasm about rhythm control for nearly two decades [5, 6]. Despite criticism of these studies with regards to the group crossover rates, cessation of anticoagulation in a substantial proportion of rhythm-control subjects, and the relatively older age of the trial population, these landmark trials have shifted priorities from arrhythmia treatment to thromboembolism prevention with anticoagulation. Randomised trial of rhythm control versus rate control of AF in patients with congestive heart failure also has not shown improvement in mortality [7]. While rate control had become the primary treatment strategy, rhythm control was still widely used for symptomatic patients [8]. Given no impact on prognosis, decisions about rate control or rhythm restoration are often driven by individual preferences and considerations of quality of life. Still, recent interest in early rhythm control strategy, particularly in younger patients with first or rare episodes of AF, reemerged with new observational and randomised data. In the GARFIELD registry, earlier cardioversion was associated with lower mortality compared to those who did not receive early cardioversion [9]. In the CASTLE-AF trial, catheter ablation for AF was shown to reduce the risk of death or hospitalisation in patients with heart failure [10]. EAST-AFNET trial had shown improved cardiovascular outcomes with rhythm control in early treatment of AF in patients with concomitant cardiovascular conditions [11]. Moreover, the advent of radiofrequency ablation and the results of new trials made rhythm control strategy more appealing again [12]. Most data on treating AF in real-world settings comes from academic centres [13], while most patients are still treated for AF within the primary care community settings. Reports on managing AF in primary care and non-academic settings are limited [14, 15] . Thus, our study aims to present data on rhythm control with electrical cardioversion in a community hospital in Russia.

## Methods

### Settings and procedure

Tarusa Hospital is a small community hospital serving nearly 15,000 people living in Tarusa and adjacent villages. It is the only hospital in the area providing primary care and basic inpatient services with two intensive care beds. Both internists and cardiologists provide cardiology care. There is universal state insurance coverage in Russia, and treatment in state facilities is free for patients.

Cardioversions are performed with standard monitoring, oxygen delivery via the face mask under sedation with propofol delivered by an internist or cardiologist. A biphasic defibrillator is used with specific discharge energies left to the physician’s discretion. Synchronised shock is delivered with hand-held paddles in anteral-lateral positions of electrodes. Anticoagulation management is performed according to guidelines, with paroxysms of less than 48 hours cardioverted without mandatory prior anticoagulation and longer paroxysms cardioverted either with previous long-term anticoagulation (of at least four weeks) or following exclusion of left atrial thrombus with transesophageal echocardiography (TEE).

Outpatient procedure usually takes one to two hours, including post-procedure monitoring of 30 to 60 minutes. If a TEE is performed before cardioversion or an antiarrhythmic drug is given between repeated shocks, the total time spent in the hospital is two to four hours. The performance of cardioversion in outpatient or inpatient settings depends on physician and patient preferences. All records regarding procedures, consultations, and hospitalisations are stored in an electronic health record (EHR) system. Additionally, a stand-alone digital database of all echocardiographies performed is maintained.

### Data

Two independent databases were used to extract the data: an EHR database and a separate digital database of all echocardiographies performed from 1 January 2016.

All records containing words specific to a description of electrical cardioversion procedures were selected from the EHR system from January 1, 2016, till December 1, 2022. Search used the following terms: “cardioversion”, “propofol”, “joule”, and “sedation”. Additionally, all TEE studies from the echocardiography archive were searched for studies performed before planned electrical cardioversion to reveal procedures not found by EHR search and assess the probability of cardioversion postponement based on TEE results. For every medical record describing electrical cardioversion, data were retrieved manually on the number of shocks, their amplitude, the immediate success of the procedure (defined as sinus rhythm at the moment of discharge for outpatients or at the end of the day for inpatients), a dose of propofol, inpatient or outpatient settings, the performance of TEE before cardioversion, chronic anticoagulation before the procedure (defined as anticoagulation for four weeks or more or till previous cardioversion), a drug used for chronic anticoagulation, an antiarrhythmic drug used adjunctive to the procedure, duration of arrhythmia episode (categorised as less than 48 hours, less than a week or unknown), any complications, date of the procedure, sex, age of the patient at the day of the procedure.

Additionally, data on medical history, including comorbidities – arterial hypertension, diabetes mellitus, coronary heart disease, heart failure – and any previous cardioversions were extracted automatically by keywords from records preceding index cardioversion.

Data on body weight and height were extracted from a separate echocardiography dataset. Data on some common echocardiographic parameters related to structural heart diseases, such as left ventricle ejection fraction and indices of the end-diastolic volume of the left ventricle, left ventricular mass and left atrial volume, were also extracted from this database and matched with the main study database. In cases with more than one measure for the patient, the mean of all the measurements was taken for weight and height, and chronologically, the first values were used for echocardiographic parameters. In cases where measurements were made with different methods (like biplane or single plane left ventricular volume or ejection fraction), the one recommended by guidelines [16] was used. Automated data extraction from EHR was performed with Python, while data cleaning and statistical analyses were done in R version 4.1.3 (2022-03-10). Data and analysis codes are available in the online repository.

### Outcomes

The main effectiveness outcome was the immediate success of the procedure (defined as sinus rhythm at the moment of discharge for outpatients or at the end of the day for inpatients). Additionally, safety outcomes included any complications described in the record.

### Statistical analysis

Suitable descriptive statistics were used for variables. The study focused on the determinants of cardioversion immediate success, which were presented as success probabilities in subgroups. Odds ratios with univariate regression were also calculated but were not reported in the main text (see Supplementary material, Table S1). We assume that for practising physicians, probabilities of success of the procedure in the subgroups are much more useful than odds ratios, which can have statistical significance but still questionable clinical utility. Point estimates and 95% confidence intervals were reported without correction for multiplicity and without *p*-values [17, 18].

Multiple logistic regression model with immediate success as the outcome was evaluated with age, gender, comorbidities, arrhythmia duration, arrhythmia type, and previous cardioversions as explanatory variables. Settings, inpatient or outpatient, were not included in the regression model because they can be influenced by cardioversion success: some patients were hospitalised due to cardioversion failure. Echocardiographic parameters (including body mass index extracted from the echocardiography database) were not evaluated in regression model because they were available only for a subset of patients with a limited number of cardioversion failures. The regression results are presented as odds ratios with confidence intervals; *p*-values are provided for convenience and do not refer to hypotheses testing.

Sensitivity analysis was not performed. Missing data was not imputed. All analyses were made for complete cases only. Sample size and power calculations were not performed as analyses were focused on all available data [19].

The study protocol was approved by the ethical committee of OOO AVA-PETER (approval number 02/23) and was registered on clinicaltrials.gov (NCT06151132).

## Results

A total of 1,380 records corresponding to the search criteria were extracted (see Supplementary material, Fig. S1). Of them, 130 were excluded due to no cardioversion described in the record, cardioversion or defibrillation for non-AF arrhythmia (regular supraventricular tachycardia, ventricular tachycardia, and ventricular fibrillation), and duplicate records describing the same procedure. One record was excluded due to missing a valid date of birth. Twenty two additional records describing cardioversions but not corresponding to search criteria were found during the analysis of the found records and TEE studies. All the records were analysed, and prespecified data were extracted.

In the study period, 1,272 procedures were performed on 435 patients, with a median (IQR) 1 (1–3) procedures per patient. Among 435 patients, 51% were female, with a median age of 66. Thirty seven percent had heart failure, and 48% had arterial hypertension. Data on weight and height extracted from the echocardiography database were available only in 239 patients, while echocardiographic parameters of interest were available for 56 to 134 patients due to incomplete echocardiography reports. Patient characteristics are provided in Table 1.

**Table 1:** Baseline characteristics of patients

		Overall, N = 435	Success^a^, N = 377	Failure^a^, N = 58
	N	Value^b^	Value^b^	Value^b^
**Demographics**				
Age (years)	435	66 (60, 76)	66 (60, 76)	68 (59, 76)
Sex	435			
Male		212 (49%)	182 (48%)	30 (52%)
Female		223 (51%)	195 (52%)	28 (48%)
**Comorbidities**				
Arterial hypertension	435	210 (48%)	181 (48%)	29 (50%)
Coronary heart disease	435	83 (19%)	69 (18%)	14 (24%)
Heart failure	435	163 (37%)	144 (38%)	19 (33%)
Diabetes mellitus	435	44 (10%)	42 (11%)	2 (3.4%)
No comorbidities	435	134 (31%)	114 (30%)	20 (34%)
**Echocardiographic parameters**^c^				
LV ejection fraction, %	135	53 (42, 61)	53 (41, 60)	60 (48, 65)
Index of LV end-diastolic volume, ml/m²	127	49 (37, 62)	50 (37, 61)	47 (33, 71)
Index of left atrial volume, ml/m²	56	41 (33, 54)	41 (32, 55)	45 (36, 49)
Index of LV mass, g/m²	110	94 (80, 124)	94 (79, 124)	95 (81, 117)
Body mass index	239	29.3 (26.3, 33.8)	29.3 (26.0, 33.7)	30.5 (27.2, 35.4)

^a^Outcomes of the first cardioversion for each patient

^b^Median (IQR); n (%)

^c^Echocardiography parameters are available only for the proportion of patients

Most procedures (87%) were performed in outpatient settings. The median (IQR) dose of propofol was 80 (60–100) mg. In 88% of cases, a single shock was delivered, two in 6.9%, and three or more shocks in 4.9%. The median shock energy was 150 (IQR 150–150) Joules. TEE to exclude left atrial thrombus was performed before 25% of procedures. There were 1,095 (86%) cardioversions for atrial fibrillation and 177 (14%) for atrial flutter. More than half of the procedures were performed for AF of less than one-week duration. The length of the hospital stay for inpatient procedures was not reported because hospitalisations were mostly due to causes unrelated to the procedure. Inpatient settings were associated with older age, female sex, coronary heart disease and heart failure. Characteristics of cardioversions are provided in Table 2.

**Table 2:** Characteristics of cardioversion procedures

	**N = 1,272**^a^
**Procedure characteristic**	
Outcome	
Sinus rhythm restored	1,173 (92%)
Complications	13 (1.02%)
Number of shocks	
1	1,122 (88%)
2	88 (6.9%)
3 or more	62 (4.9%)
First cardioversion	358 (28%)
**Setting and speciality of provider**	
Care settings	
Outpatient	1,103 (87%)
Inpatient	169(13%)
Performed by	
Internist	403 (32%)
Cardiologist	869 (68%)
TEE performed	323 (25%)
**Arrhythmia characteristics**	
Type of arrhythmia	
AF	1,095 (86%)
Atrial flutter	177 (14%)
Arrhythmia less than 48 hours	606 (48%)
Arrhythmia less than 7 days	783 (62%)
Arrhythmia duration unknown	198 (16%)
**Drugs used before, during and after procedure**	
Prior anticoagulation	
Warfarin	218 (17%)
Direct oral anticoagulant	661 (52%)
None	393 (31%)
Antiarrhythmic drug used during cardioversion	
None	1,188 (93%)
Amiodarone	75 (5.9%)
Other	9 (0.7%)
Rhythm and rate control drugs prescribed after cardioversion	
Beta-blocker	617 (49%)
None	280 (22%)
Amiodarone	109 (8.6%)
Sotalol	213 (17%)
Propafenone	19 (1.5%)
Verapamil	31 (2.4%)
Diltiazem	3 (0.2%)

^a^n (%)

Rhythm control drugs, namely amiodarone, sotalol, and propafenone, were prescribed after the cardioversion procedure in 8.6%, 17%, and 1.5% of cases. Beta-blockers were prescribed after 49% of procedures, and in 22%, neither rhythm control nor rate control drugs were prescribed. Rhythm control drugs were prescribed at least once after cardioversion to 29% of patients.

Periprocedural administration of antiarrhythmic drugs was performed in 5.9% of procedures, and in most cases, it was amiodarone. Sixty-nine percent of patients were chronically anticoagulated before cardioversion, including 17% on warfarin and 52% on direct oral anticoagulants. Details are provided in Table 2.

A total of 379 TEE studies were performed before planned electrical cardioversions, with 56 of them resulting in declined cardioversions: 30 due to left atrial thrombus, 12 due to left atrial sludge or suspicion of thrombus, six due to failure to intubate, four due to spontaneous restoration of sinus rhythm and four due to other reasons.

### Effectiveness

Sinus rhythm was restored in 1,173 (92%) procedures. The success probability was higher than 90% in most subgroups (Table 3). In the multiple regression model, factors independently associated with cardioversion failure were duration of arrhythmia more than seven days (OR 0.50, 95% CI 0.31-0.79) and no prior cardioversions (OR 0.47, 95% CI 0.29-0.75) (Table 4).

**Table 3:** Cardioversion success probabilities in subgroups

		N	Probability, %	95% CI^a^
**Patient characteristics**				
Age	Older than 65 years	692	92	90–94
	65 years or younger	580	92	90–94
Sex	Male	628	92	90–94
	Female	644	92	90–94
Heart failure	Yes	565	92	90–94
	No	707	92	90–94
Arterial hypertension	Yes	741	93	91–95
	No	531	91	89–94
Coronary heart disease	Yes	268	94	90–96
	No	1004	92	90–93
Diabetes mellitus	Yes	174	95	91–98
	No	1098	92	90–93
Body mass index	More than 30	372	94	91–96
	30 or less	406	92	89–95
LV ejection fraction	Less than 60%	315	93	89–95
	60% or more	162	**88**	**81–92**
Left atrial index	Above the median	143	92	86–96
	Median or below	161	94	89–97
**Arrhythmia characteristics**				
Arrhythmia duration	More than 7 days	489	**88**	**85–91**
	7 days or less	783	95	93–96
Type of arrhythmia	Atrial fibrillation	1095	92	90–94
	Atrial flutter	177	93	88–96
First cardioversion	Yes	358	**86**	**82–90**
	No	914	95	93–96
**Procedure characteristics**				
Settings	Outpatient	1103	94	92–95
	Inpatient	169	**82**	**75–87**
Performed by	Cardiologist	869	92	90–94
	Internist	403	93	89–95
TEE before procedure	Performed	323	**89**	**85–92**
	Not performed	949	93	91–95

^a^CI – confidence interval

**Table 4:** Factors associated with cardioversion success in multiple logistic regression model

Characteristic	OR^a^	95% CI^a^	*p*-value
Age > 65 years	0.87	0.56-1.36	0.6
Men (vs. Women)	1.08	0.68-1.71	0.7
Heart failure	0.97	0.63-1.50	0.9
Arterial hypertension	0.90	0.56-1.44	0.7
Coronary heart disease	1.29	0.75-2.33	0.4
Diabetes mellitus	1.83	0.90-4.26	0.12
Arrhythmia > 1 week	0.50	0.31-0.79	0.003
Artial fibrillation (vs. flutter)	0.79	0.39-1.44	0.5
First cardioversion	0.47	0.29-0.75	0.002

^a^OR = Odds Ratio, CI = Confidence Interval

### Complications

There were 13 complications reported (1% of procedures). Two complications required hospitalisation for one day, one patient was hospitalised for a longer period, but hospitalisation was not related to complications, and three complications occurred in patients who had already been hospitalised. Most complications were related to sedation and did not require hospitalisation; one complication was related to antiarrhythmic drugs in patients hospitalised for heart failure. Hospitalisations were mostly related to preexisting heart failure. A detailed description of complications is provided in Table 5.

**Table 5:** Complications

no.	Description of event	Rhythm restored	Hospitalisation	Sex, age
1	Agitation on propofol, electrical cardioversion canceled, rhythm restored the next day by amiodarone infusion.	No	Yes (1 day)	Male, 57
2	Oversedation and transient hypoxemia requiring bag-mask ventilation.	Yes	Already hospitalised for heart failure.	Female, 60
3	Oversedation, transient hypoxemia (SpO2 75%).	Yes	Already hospitalised for heart failure	Female, 77
4	Arterial hypotension on amiodarone infusion, hospitalised due to heart failure.	Yes	Hospitalised the same day for heart failure	Female, 77
5	Oversedation and transient hypoxemia (SpO2 82%) during TEE, requiring head tilt and chin Lift.	Yes	No	Female, 69
6	Oversedation, patient in alcohol intoxication, propofol dose 100 mg.	Yes	No	Male, 41
7	Oversedation, propofol dose 60 mg.	Yes	No	Female, 80
8	Oversedation and transient hypoxemia during TEE, exam aborted.	Yes	No	Female, 72
9	Arterial hypotension, bradycardia, and syncope after ambulation.	Yes	Already hospitalised for myocardial ischemia	Male, 82
10	Protracted arterial hypotension (80/60 mm Hg) without shock after cardioversion.	Yes	Yes (1 day)	Male, 53
11	Long pause after cardioversion (less than a minute), few chest compressions delivered, atrial rhythm ensued, and later, a pacemaker was implanted for recurring post-cardioversion pauses. The patient was shortly after cardiac surgery for mitral regurgitation.	Yes	No	Male, 64
12	Supraventricular tachycardia ensued after cardioversion, terminated with transesophageal overdrive pacing.	Yes	No	Female, 79
13	Transient bradycardia (escape rhythm with a heart rate of 45/min).	Yes	No	Female, 78

### Patient-centered outcomes

Being translated into patient-centred language, our results could be summarised as follows: electrical cardioversion is quite a safe procedure that, in most cases, can be performed in outpatient settings; it restores sinus rhythm in nearly 9 out of 10 patients with complications in nearly 1 out of 100 patients, of these most common being transient low blood pressure or breathing pauses with low blood oxygen not requiring any interventions or hospitalisation.

## Discussion

Our data show high immediate effectiveness of direct current cardioversion similar to other studies [20, 21] with low risk of serious complications. This procedure was safe, with no serious complications in over a thousand cases. In most cases, it could be performed in outpatient settings, with patients discharged home in one or two hours. Its effectiveness is more than 90%, meaning sinus rhythm will be restored in 9 out of 10 patients. Interestingly, in all registries, we have found men were predominant in patients undergoing electrical cardioversion (73.2% in [20], 64.4% in [9], more than 70% in [22], 68% in [21]) while in our study there were almost equal numbers of men and women. Most of the procedures were performed in outpatient settings, and no procedure-related factors were associated with inpatient settings except for the duration of arrhythmia and heart failure. Hospitalisations were not related to cardioversion *per se* but were related to other indications, mostly heart failure and social factors. Overall, TEE was performed before nearly every fifth procedure in outpatient settings and every fourth in inpatient settings. The availability of TEE allows procedures to be performed on the day of presentation, which is more convenient for patients.

While the effectiveness of the cardioversion differed in inpatient and outpatient settings, we do not consider settings as a predictor of success not only because of confounding of age and comorbidities, which can be adjusted for, but also because failure to restore sinus rhythm can cause hospitalisation.

The association between longer duration of atrial fibrillation and cardioversion failure, although not universally observed, has been shown in some previous studies [23]. It can be explained by atrial remodelling, the presumed mechanism of atrial fibrillation self-perpetuation [3]. The greater effectiveness of cardioversion in repeat procedures compared to the first procedure has not been reported in other studies. In fact, in the RHYTHM-AF Registry data from Poland, the first cardioversion was associated with greater success [20]. A possible explanation is a selection based on some unobserved patient factors: repeated cardioversions were not performed if the first cardioversion was unsuccessful due to these factors, and a rate control strategy was pursued, whereas in patients prone to successful cardioversion procedures were repeated for subsequent arrhythmia recurrences.

A cardiologist or internist performed the procedure. No anesthesiologist was present for sedation. In Russia, this procedure is traditionally performed in inpatient settings by cardiologists and anesthesiologists, hospitalisation being a barrier to the wider use of this procedure, especially in rural areas and community hospitals, where this procedure is underused. While cardioversion service is led by nurses in some countries, [24–26] in Russia, nurses perform only a few minor procedures independently.

Compared to other studies, fewer antiarrhythmic drugs were prescribed to patients in our study. Both in AFFIRM and EAST-AFNET 4 trials comparing rate and rhythm control strategies, significantly more than half of the patients in the rhythm-control arm were receiving antiarrhythmic drugs despite higher availability of radiofrequency ablation in the latter trial [6, 11]. In the Canadian registry, 41% of patients were on rhythm control medication and 12.3% on beta-blockers after diagnosis of AF [27]. In our study, rhythm control drugs were prescribed only after 27% of procedures, and only 29% of patients were prescribed any rhythm control drug at least once after cardioversion. The majority of the patients received no rhythm control drugs. Beta-blockers were prescribed after almost half of the procedures to prevent fast ventricular response during further paroxysms or for other indications. This seems reasonable considering readily available access to cardioversion service, controversies about antiarrhythmics conferring mortality risk, and difficulties in assessing their effectiveness when paroxysms of arrhythmia are infrequent.

Our study has several limitations. While our hospital is the only hospital in the area, some patients from neighbouring districts receive care here, and some patients from our district can be treated in other places, like tertiary hospitals in the regional capital or Moscow. Still, we are the only facility in the broader region where electrical cardioversion is available in the outpatient settings, which increases the chances that patients will be referred and followed up in our hospital. Also, our study was performed in a single center and did not represent common practice in other community hospitals in Russia. As far as we know, electrical cardioversion for AF is scarcely used in most other hospitals in our region and is rarely done in outpatient settings in this country because of reimbursement issues, logistics, tradition, or legal concerns.

An important limitation is related to the retrospective nature of our study and the use of EHR as the main data source. While EHR greatly facilitates the collection and analysis of medical data, the reliability of this data fully depends on the accuracy of physicians and nurses entering them. Templates with required fields ensure the completeness of the data, but the use of copy-paste makes it much less reliable [28]. In patients with repeated procedures, records tend to be too short, like “cardioversion performed as usual under propofol, patient discharged in sinus rhythm on same medications”.

Another limitation of our study is the absence of data on CHA(2)DS(2)-VASc and HAS-BLED scores, so we could not assess whether anticoagulation was administered according to guidelines. Data on cardioversion’s long-term effects were also not analysed in this study. While presented analyses are useful for practising physicians in assessing the immediate effects of the procedure, longer-term outcomes, like time in sinus rhythm, are also important. This study will be extended to capture the long-term effects of cardioversion.

Our study also has some strengths. It is performed in community settings, which enables it to inform practice in other community hospitals. All consecutive electrical cardioversions were included in the analysis, which makes selection bias less probable. Unlike in other AF and electrical cardioversion registries, there was an equal number of men and women in our study. It informs physicians and policymakers and directly answers the questions that can arise in patients with AF about the procedure.

While EHR provides the opportunity to perform retrospective studies and assess clinical practice in primary care and community settings, many interesting and possibly important data are missing. This can be avoided in prospective studies and dedicated registries, but these are often performed in academic centres and tertiary clinics, so they are not always generalisable. Possibly, some research framework should be established to make participation in clinical research more readily available to physicians and nurses in community settings, as it was proposed more than a century ago [29].

In conclusion, electrical cardioversion is part of AF rhythm control strategy treatment. It is an immediately effective procedure that can be safely performed in community hospitals in outpatient settings. Collecting and analysing data on performed treatments is important to inform patients during shared decision-making. This study will be supplemented by the analysis of long-term outcomes, which would add additional value to this project.

### Supplementary Information

**Additional file 1.** Supplementary material with the additional figures and tables.

## Data Availability

Data is publicly available at OSD repository: https://osf.io/xpj4z.
