# Supplementary material for: Atrial fibrillation: real-life experience of a rhythm control with electrical cardioversion in a community hospital
Source: BMC Cardiovasc Disord. 2024 Apr 17;24:213. doi: 10.1186/s12872-024-03885-0 (PMC11022487; doi:10.1186/s12872-024-03885-0)
Supplement: Supplementary file 1 — Additional file 1. Supplementary material with the additional figures and tables. [file 12872_2024_3885_MOESM1_ESM.pdf]

Supplementary material

Atrial fibrillation: real-life experience of a rhythm control with electrical cardioversion in a community hospital

Artemiy Okhotin, Maxim Osipov, Vasilij Osipov, Anton Barchuk

Figure S1: Flowchart illustrating acquisition of data on cardioversions.
Table S1. Univariate predictors of successful cardioversion.
Table S2. First cardioversion success probabilities in subgroups.
Table S3. First cardioversion success probabilities in subgroups.

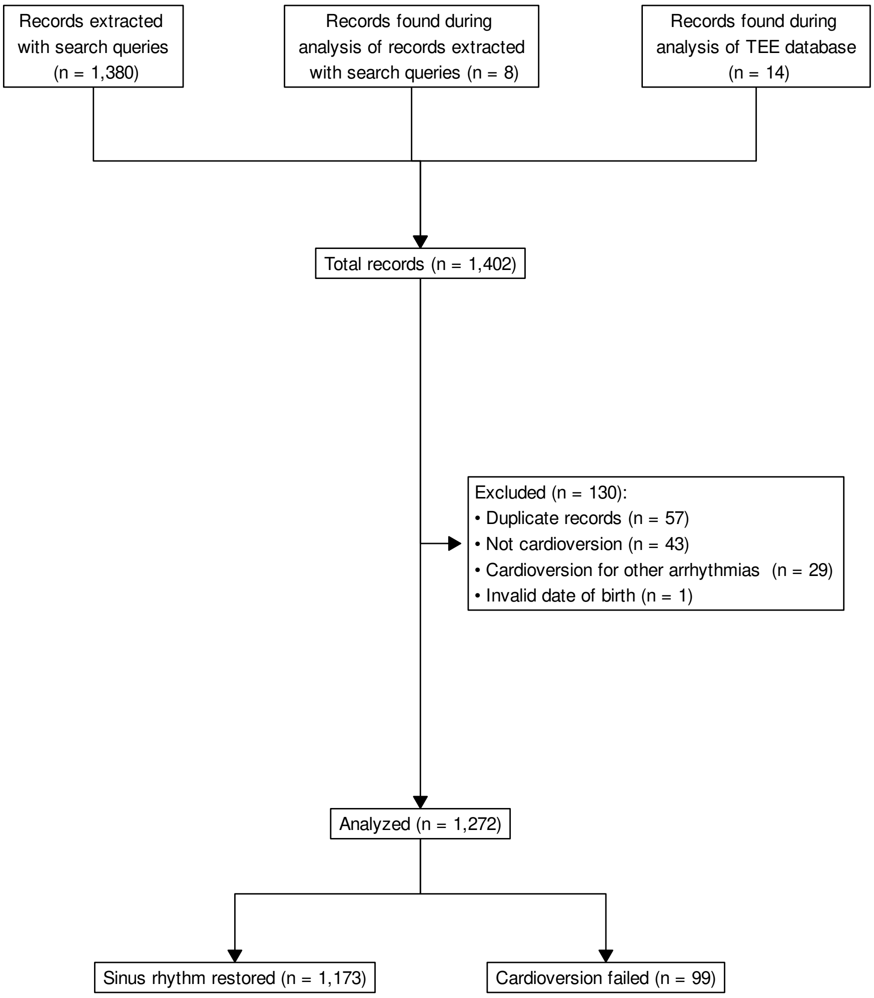

Figure S1: Flowchart illustrating acquisition of data on cardioversions.

Table S1: Univariate predictors of successful cardioversion.

Characteristic	OR	95% CI	p-value
Age > 65 years	0.95	0.63–1.43	0.8
Men (vs. Women)	1.04	0.69–1.57	0.9
Heart failure	1.00	0.66–1.52	> 0.9
Arterial hypertension	1.23	0.81–1.86	0.3
Coronary heart disease	1.31	0.78–2.33	0.3
Diabetes mellitus	1.88	0.95–4.26	0.10
Arrhythmia > 1 week	0.39	0.26–0.59	< 0.001
Artial fibrillation (vs. flutter)	0.84	0.43–1.52	0.6
First cardioversion	0.39	0.26–0.59	< 0.001
Outpatient (vs. inpatient)	3.42	2.14–5.38	< 0.001
Performed by cardiologist (vs. internist)	0.93	0.59–1.44	0.8
TEE performed	0.60	0.39–0.93	0.019

[1] OR = Odds Ratio, CI = Confidence Interval

Table S2: First cardioversion success probabilities in subgroups.

		N	Probability, %	95% CI[1]
**Patient characteristics**				
Age	Older than 65 years	182	84	77–88
	65 years or younger	176	89	83–93
Sex	Male	185	87	81–92
	Female	173	86	81–91
Heart failure	Yes	124	87	80–92
	No	234	86	81–90
Arterial hypertension	Yes	142	85	78–90
	No	216	87	82–91
Coronary heart disease	Yes	52	85	71–93
	No	306	87	82–90
Diabetes mellitus	Yes	34	94	79–99
	No	324	85	81–89
Body mass index	More than 30	90	87	77–93
	30 or less	113	89	82–94
LV ejection fraction	Less than 60%	88	93	85–97
	60% or more	32	72	53–86
Left atrial index	Above the median	22	91	69–98
	Median or below	24	88	67–97
**Arrhythmia characteristics**				
Arrhythmia duration	More than 7 days	237	85	80–89
	7 days or less	121	88	81–93
Type of arrhythmia	Atrial fibrillation	299	86	81–90
	Atrial flutter	59	88	76–95
Settings	Outpatient	265	89	85–93
	Inpatient	93	77	67–85
Performed by	Cardiologist	267	85	80–89
	Internist	91	89	80–94
TEE before procedure	Performed	221	87	82–91
	Not performed	137	85	78–91

[1] CI – confidence interval.

Table S3: Factors associated with inpatient settings

**Characteristic**	**N**	**OR**	**95% CI**
Age (years)	1,272	1.04	1.02—1.05
Sex	1,272		
Male			
Female		1.37	0.99—1.90
Arterial hypertension	1,272	0.62	0.45—0.85
Coronary heart disease	1,272	1.83	1.27—2.60
Heart failure	1,272	2.66	1.90—3.74
Diabetes mellitus	1,272	0.44	0.23—0.78
TEE performed	1,272	4.13	2.96—5.78
Arrhythmia more than 7 days	1,272	2.98	2.14—4.18
Chronic anticoagulation	1,272	0.25	0.18—0.35
Type of arrhythmia	1,272		
Atrial Fibrillation			
Atrial Flutter		0.97	0.59—1.53
Body mass index	778	0.99	0.96—1.02
LV ejection fraction, %	477	0.97	0.95—0.98
Index of left atrial volume, ml/m^2	304	1.01	0.98—1.03

[1] OR = Odds Ratio, CI = Confidence Interval
